# Genome diversification within a clonal population of pandemic *Vibrio parahaemolyticus* seems to depend on the life circumstances of each individual bacteria

**DOI:** 10.1186/s12864-015-1385-8

**Published:** 2015-03-13

**Authors:** David E Loyola, Cristell Navarro, Paulina Uribe, Katherine García, Claudia Mella, Diego Díaz, Natalia Valdes, Jaime Martínez-Urtaza, Romilio T Espejo

**Affiliations:** Centro Nacional de Genómica y Bioinformática, Av B. O’Higgins 340, Santiago, Chile; Instituto de Nutrición y Tecnología de los Alimentos, Universidad de Chile, El Líbano 5524, Santiago, Chile; Depatment of Biology and Biochemistry, University of Bath, Claverton Down, Bath, North East Somerset BA2 7AY UK

**Keywords:** *Vibrio parahaemolyticus*, Evolution, Genome innovation, Horizontal gene transfer, Single nucleotide variants

## Abstract

**Background:**

New strains of *Vibrio parahaemolyticus* that cause diarrhea in humans by seafood ingestion periodically emerge through continuous evolution in the ocean. Influx and expansion in the Southern Chilean ocean of a highly clonal *V. parahaemolyticus* (serotype O3:K6) population from South East Asia caused one of the largest seafood-related diarrhea outbreaks in the world. Here, genomics analyses of isolates from this rapidly expanding clonal population offered an opportunity to observe the molecular evolutionary changes often obscured in more diverse populations.

**Results:**

Whole genome sequence comparison of eight independent isolates of this population from mussels or clinical cases (from different years) was performed. Differences of 1366 to 217,729 bp genome length and 13 to 164 bp single nucleotide variants (SNVs) were found. Most genomic differences corresponded to the presence of regions unique to only one or two isolates, and were probably acquired by horizontal gene transfer (HGT). Some DNA gain was chromosomal but most was in plasmids. One isolate had a large region (8,644 bp) missing, which was probably caused by excision of a prophage. Genome innovation by the presence of unique DNA, attributable to HGT from related bacteria, varied greatly among the isolates, with values of 1,366 (ten times the number of highest number of SNVs) to 217,729 (a thousand times more than the number of highest number of SNVs).

**Conclusions:**

The evolutionary forces (SNVs, HGT) acting on each isolate of the same population were found to differ to an extent that probably depended on the ecological scenario and life circumstances of each bacterium.

**Electronic supplementary material:**

The online version of this article (doi:10.1186/s12864-015-1385-8) contains supplementary material, which is available to authorized users.

## Background

*Vibrio parahaemolyticus* is an autochthonous ocean-dwelling microbial species comprising bacterial strains that are widely disseminated in marine environments throughout the world. Some of these strains cause severe diarrhea when present in seafood [1]. New isolates of *V. parahaemolyticus* that are able to cause diarrhea in humans by ingestion of seafood periodically emerge through their continuous evolution in the ocean. This process occurs via mutations and horizontal gene transfer. The epidemiology of the diarrhea outbreaks caused by different *V. parahaemolyticus* serotypes changed abruptly after 1996, when the emergence of a particular clonal strain (serotype O3:K6) increased the number of diarrhea outbreaks in Southeast Asia initially, and subsequently worldwide [2,3]. It is likely that this strain acquired pathogenicity islands [4] and secretion system [5] genes by lateral transfer, thereby increasing its pathogenicity. Arrival of this strain (called pandemic *V. parahaemolyticus*) in a region of southern Chile where most of this country’s seafood is harvested caused one of the world’s largest outbreaks of seafood-related diarrhea [6,7]. The food poisoning outbreaks peaked in 2005 with about 10,000 clinical cases, and then gradually decreased to fewer than 100 cases in 2010 to 2013 (Detailed information on the epidemiology of *V. parahaemolyticus* in Chile can be found in the web page of the ministry of Health, http://epi.minsal.cl/vigilancia-epidemiologica/enfermedades-de-notificacion-obligatoria/vibrio-parahaemolyticus/). It is though that after its arrival, the pandemic strain growth was favored by both a minor rise in surface seawater temperature and warming of the mussels in the intertidal region due to frequent sunny days. Later, a selective effect of bacteriophages with different tropism could have caused its decline [7].

Humans become infected when the bacteria occasionally reach the human gastrointestinal tract, mainly via consumption of raw or undercooked seafood. During the Chilean epidemic, the pandemic strain consisted of a highly clonal population [8], which reproduced extensively in the Chilean shore lines, and shared its habitat with many other related species. It was calculated that mussels on the shores from which these isolates were obtained contained up to 62 different *V. parahaemolyticus* genotypes [7].

Here, we investigated the degree of genomic diversification that occurred in this homogeneous population of bacteria during a short independent divergence time among the isolates. Previous studies performed by comparison of different DNA sequences such as housekeeping genes and multiple tandem repeats, and more recently whole genome sequencing, have shown a small degree of genetic diversity between isolates obtained throughout the world [8-11]. In terms of studying bacterial evolution in a natural habitat, the pandemic *V. parahaemolyticus* population appears to be particularly appropriate. As Achtman and Wagner [12] wrote in their review “*Bacterial pathogens with low synonymous sequence diversity (DS of <0.0002), so-called genetically monomorphic organisms, are promising models to reveal evolutionary mechanisms, and could even be more informative than microbes with greater sequence diversity, in which millions of years of evolutionary history might have blurred genomic signals of phylogenetic history through recombination or eliminated them through genomic reduction*”.

In this work, we obtained eight independent Chilean isolates of *V. parahaemolyticus* from mussels or clinical samples and compared their genomes. We show that the predominant evolutionary forces acting on the isolates differ and are probably dependent on the ecological situation and the life circumstances of each bacterium. Many of the changes detected would have been difficult to detect in a more heterogeneous bacterial population with longer independent evolution times among its members. Thus, the *V. parahaemolyticus* pandemic population constitutes an appropriate model to study evolutionary mechanisms, despite its low synonymous sequence diversity resulting from its short evolutionary history.

## Results

The genomes of eight Chilean *V. parahaemolyticus* isolates and of isolate VpKX, a subculture of the RIMD 2210633 [13] strain from South East Asia maintained in our laboratory since its arrival from the Research Institute of Medical Disease in Japan in 2002, all corresponding to the pandemic strain group, were sequenced using both a single-end and a mate paired-ends with 3 Kb span libraries. Table 1 shows the properties of these isolates and those of VpKX and RIMD 2210633 whose genome has been previously sequenced. The Chilean isolates were chosen because some represented early isolates from the initial outbreak in 1998 (ATC210 and ATC220) and the others those obtained at the peak of the outbreak, between 2004–2007; two corresponded to isolates obtained from shellfish (PMA37.5 and PMA109.5). Two of the isolates were also chosen because they contained plasmids. Isolate PMA109.5 contains three plasmids previously observed after agarose gel electrophoresis of a plasmid extract (not shown) and isolate PMC58.5 harbors the temperate linear plasmid bacteriophage VP58.5 [14,15]. The sequencing depth coverage was between 25X to 49X for single-end reads and 5X for mate paired-end reads, except for PMA37.5, which was not sequenced using mate paired-end library (Additional file 1: Table S1).

**Table 1:** **General properties of the pandemic** ***V. parahaemolyticus*** **isolates compared in this project**

**Strain**	**Origin**	**Isolation Year**	**DGREA profile**	**Plasmids**	**Ref.**
RIMD 2210633	Southeast Asia	1996	VpKX	-	[13]
VpKX	RIMD 2210633 maintained in Chile since 2002	1996	VpKX	-	[13]
ATC210	Antofagasta, clinical	1998	VpKX	-	[6]
ATC220	Antofagasta, clinical	1998	VpKX	-	[6]
PMC48	Puerto Montt, clinical	2004	VpKX	-	[6]
PMC58.5	Puerto Montt, clinical	2005	58.5	VP58.5	[14-16]
PMA37.5	Puerto Montt, mussel	2005	VpKX		[16]
PMA109.5	Puerto Montt, mussel	2005	109.5	-VP58.5*	[16]
				~100 Kbp*	
				~90 Kbp*	
PMC14.7	Puerto Montt, clinical	2007	VpKX	-	[17]
PMC58.7	Puerto Montt, clinical	2007	VpKX	-	[17]

DGREA, direct genomic restriction enzyme analysis.

*Additional bands observed in Pulse field gel electrophoresis or in gel electrophoresis after plasmid extraction.

### Single Nucleotide Variants (SNVs)

**Figure 1:**
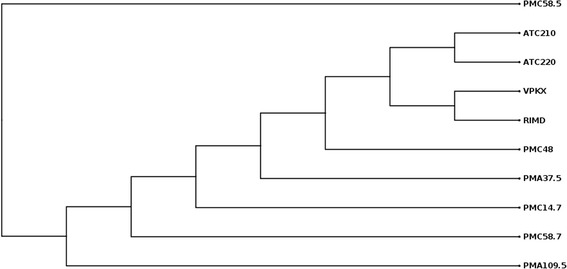
**Unrooted cladogram showing the relations of the** ***V. parahaemolyticus*** **pandemic isolates from Chile and also those of Southeast Asia, RIMD 2210633 and VpKX.** Obtained using neighbor joining algorithm with Jukes-Cantor distance measure using 1,000 bootstrap replicates.

High quality SNVs between the shared genome portion of each isolate were calculated as recently described for *V. cholerae* [18] by mapping the trimmed reads against the RIMD 2210633 genome [13] and considering only SNVs with depth coverage higher than 0.4 times the average depth of coverage for each strain (values varied from 8 to 16), and a minimum variant frequency of 75%. Deletions and insertions were not considered. Positions showing SNVs between the sequence of each isolate and RIMD 2210633 genome were identified, extracted and converted in a concatenated sequence. The resulting concatenated sequence of each isolate was compared with the sequences obtained from all the isolates. This comparison allows determining the SNVs among the panel of isolates compared. The total number of polymorphic sites (length of the concatenate) observed among isolates was 270 with 591 different differences in base composition. Figure 1 is an unrooted cladogram showing the relations of the *V. parahaemolyticus* pandemic isolates from Chile and also those of Southeast Asia, RIMD 2210633 and VpKX. The number of SNVs among the isolates varied from 13 to 164 (Table 2, above diagonal). Analysis of the distribution of SNVs along the genome showed that they had a Poisson distribution as expected for independent mutations that randomly occur in a genome (results not shown). No SNV clusters were observed, as it was by Chen et al. [11] in AN5034, a pandemic isolate that apparently changed serotype by genetic recombination with a closely related *V. parahaemolyticus* strain [11]. Twenty-nine SNVs were observed between the reported sequence of RIMD 2210633 and the sequence obtained for VpKX. However, this was not surprising because we had previously observed differences in the number of serial repeats in some multiple tandem repeat loci between VpKX and the reported sequence of RIMD 2210633 [8]. Such differences were probably generated (particularly those from the multiple repeats analysis) during the growth of the bacteria that occurred in the sealed stab agar, which was initially kept at room temperature and was renovated every six months for at least five years. After this time the strain was kept frozen.

**Table 2:** **Number of SNVs (upper triangle) and number of additional or diminished base pairs (lower triangle) between Chilean pandemic** ***V. parahaemolyticus*** **isolates and Southeast strains RIMD 2210633 and VpKX included in this study**

**Isolate**	**PMC14.7**	**PMC48**	**PMA37.5**	**PMC58.5**	**PMA109.5**	**ATC220**	**PMC58.7**	**ATC210**	**VPKX**	**RIMD**
**PMC14.7**	-	13	18	15	32	26	45	127	53	52
**PMC48**	1,498	-	17	18	35	23	50	124	54	49
**PMA37.5**	7,429	8,643	-	23	40	30	55	129	61	54
**PMC58.5**	42,642	43,856	49,787	-	31	31	48	132	58	57
**PMA109.5**	210,300	211,798	217,729	167,942	-	46	63	147	73	72
**ATC220**	5,665	7,163	12,952	48,307	215,965	-	63	115	49	44
**PMC58.7**	1,366	2,580	8,191	43,724	211,666	7,031	-	164	88	87
**ATC210**	3,938	5,152	10,703	46,296	214,238	9,603	4,700	-	150	143
**VPKX**	5807	7,021	12,952	48,165	216,107	142	6,889	9,461	-	29
**RIMD**	5,940	7,154	13,085	48,298	216,240	275	7,022	9,594	133	-

### Genome size

The genome size of each isolate was estimated as being the length of the genome covered after alignment of the isolate reads against the complete genomic sequence of RIMD 2210633, plus the sum of the length of the contigs formed by reads that did not align to the RIMD 2210633 genome. To define these contigs, unaligned reads (1% to 16% of the total reads for each isolate) were recovered and assembled. The sum of the length of these contigs was then added to the length of the RIMD 2210633 genome that was covered to estimate the size of each isolate’s genome. Only contigs larger than 316 bp that were not similar to the plasmid vectors used for the library preparation (present in the UNIVEC database at http://www.ncbi.nlm.nih.gov/tools/vecscreen/about/#aboutvecScreen) and had a coverage depth greater than 11, which corresponds to approximately ¼ of the average coverage for the whole genome, were considered. These contigs were also subsequently expanded by further assembly using CAP3. Table 3 shows the estimated genome length of the different Chilean isolate and VpKX calculated as the length of the RIMD genome plus the length of the contigs formed by reads that did not align with this genome. The length of the RIMD genome covered by the reads of all the isolates would correspond to the core genome of the group. The genome size differences relate to the presence or absence of DNA regions in some of the isolates but some isolates pair having the same genome size may have gained or lost different DNA regions. The amount of DNA gained or lost between pairs (Table 2, below diagonal) was calculated as the sum of the length of the regions present in one isolate but absent in the other and is shown in Table 2, under the diagonal. It is not possible to determine if these regions correspond to either newly acquired DNA or inherited DNA lost by the ancestral bacterium. The length of the largest segment in the RIMD genome not covered by reads amounted to 8,787 bp and corresponded to isolate PMC48. However, we do not imply that this corresponds to DNA lost because the RIMD 2210633 strain may not be an ancestral bacterium in the Chilean isolates.

**Table 3:** **Genome characteristics of Chilean pandemic** ***V. parahaemolyticus*** **isolates and Southeast Asia strains RIMD 2210633 and VpKX**

**Isolate**	**Length of RIMD 2210633 covered with aligned reads**	**Sum of the length of contigs formed by reads not aligning with RIMD 2210633**	**Estimated size of the genome**
RIMD 2210633*	-	0	5,165,770
VpKX	5,165,637	0	5,165,637
ATC210	5,165,621	6,390	5,172,011
ATC220	5,165,621	0	5,165,621
PMC48	5,156,993	5,868	5,162,861
PMC58.5	5,165,610	48,413	5,214,023
PMA37.5	5,165,600	13,858	5,179,458
PMA109.5	5,165,584	216,349	5,381,933
PMC14.7	5,165,618	10,814	5,176,432
PMC58.7	5,164,546	6,304	5,170,850

*Data from reference [13].

The nature of the genomic regions that were gained or lost was determined using different procedures. Calculation of their GC content (Table 4) showed large differences with that of RIMD 2210633, which have an average GC content of 45.4%. Annotation using the basic local alignment search tool (BLAST, (http://blast.ncbi.nlm.nih.gov/Blast.cgi) against the National Center for Biotechnology Information (NCBI) non-redundant nucleotide database, showed similarity with regions annotated in related species but no pathogenic or secretion related regions were identified. Regions not covered by the reads were annotated for the respective regions from the whole genome description of RIMD 2210633 [13]. These final regions correspond only to segments longer than five neighbor bases with zero coverage after aligning the reads against the reference genome using two different procedures: Tmap/SAMtools and CLC workbench. No isolates, including VpKX, had coverage of two regions of 14 and 119 bp each in the reference genome of RIMD 2210633. Because we compared only Chilean isolates these two segments were not considered further. The presence or absence and the properties of the genomic regions in the Chilean isolates is shown in Table 4. No virulence factors were evident when annotating the contigs absent in RIMD 2210633. Three regions were inserted (5,665 bp, 6,637 bp, and 160/384 bp). Their insertional nature and position was determined by detection of sequences homologous to the reference genomes at the ends of the scaffolds and reads containing part of the sequence of the insert, together with part of the reference genome sequence. One of the inserts corresponds to an insertion sequence (IS) of 5,665 nucleotides present in every Chilean isolate, except ATC220, which has also been reported in three other pandemic *V. parahaemolyticus* isolates obtained from Bangladesh, Peru and India [11]. This IS is flanked by 18 bp direct repeats that are identical to the attachment site (*attR*) described in the defective but excisable lambdoid prophage CP-457 [19], and is inserted in tandem with another IS located next to a tmRNA in the South East Asian reference isolate genome [13]. Part of the IS shows high similarity with a phage integrase and a mobile element protein found in *V. parahaemolyticus* UCM-V493 [20], a strain isolated from a Spanish sediment sample that is closely related to RIMD 2210633. A second insert exclusive to PMA37.5 consists of a 6,637 bp element flanked by a 4 bp repeat (TTCG), which contains segments annotated as a phage integrase and a putative transposase in *V parahaemolyticus* UCM-V493 [20]. The whole region is highly similar to regions found in *V parahaemolyticus* UCM-V493, *V. anguillarum* 775 [21] and *V. cholerae* MJ1236 [22] genomes. A third putative IS found in three of our isolates but potentially inserted into only two isolates at different positions, consists of a isolate-specific region of variable size (393 to 563 bp), containing 5 to 12 tandem repeats.

**Table 4:** **Genome comparison of Chilean pandemic** ***V. parahaemolyticus*** **isolates**

**Segment**	**Properties**	**ATC 220**	**PMC 14.7**	**PMC 48**	**PMA 37.5**	**PMC 58.5**	**PMA 109.5**	**PMC 58.7**	**ATC 210**
	**Regions formed by reads unaligned with RIMD 2210633**								
5,665 bp Insert	Insertion Sequence in position 674,415 of RIMD 2210633. Similarity with phage integrase and mobile element protein found in *V. parahaemolyticus* UCM-V493 [21]. GC% = 36.7. Average GC% in RIMD 2210633 is 45.4%	-	P	P	P	P	P	P	P
6,637 bp Insert	6,637 bp inserted in position 2.803.591 of RIMD 2210633. Closely related with regions found in other *Vibrio* species. With segments assigned to CDS designated as phage integrase and putative transposase. GC% = 41.5	-	-	-	P	-	-	-	-
160/384 bp Inserts	5 to 12 tandem repeats with 32 bp units potentially inserted in two isolates at different positions. GC% = 50.0	-	-	-	P	-	-	P	P
Prophage VP58.5	Lineal plasmid prophage of temperate bacteriophage VP58.5 [14,15]. GC% = 50.9	-	-	-	-	P	P	-	-
82.0 Kb plasmid	83% coverage with 80% identity to plasmid p0908, a putative prophage also found in a *Vibrio* sp. [23]. GC% = 45.1	-	-	-	-	-	P	-	-
85.8 Kb plasmid	97% coverage with 99% identity to plasmid pVPUCMV of environmental strain *V. parahaemolyticus* UCM- V493 [21]. It might be a prophage but only a few ORFs are annotated. GC% = 40.8	-	-	-	-	-	P	-	-
361 bp contig	327 bp 91% identity *pvd*J in drug resistant Ps. Aeruginosa PA381812. GC% = 49.0	-	-	-	P	-	-	-	-
	**Regions in RIMD 2210633 not covered by reads from at least one other isolate**								
Chromosome 1*									
396,525–396,578	16/93 bp without annotation in reference genome	-	-	P	P	P	-	P	P
670,436–670,451	16 bp VP0639 Hypothetical	P	P	P	P	P	P	P	-
671,192–671,222	31 bp without annotation in reference genome	P	P	P	P	P	P	P	-
1,658,835–1,667,479	8644 bp in PMC48 corresponding to VP1575-VP1583, genes of prophage f237	P	P	-	P	P	P	P	P
1,674,168–1,677,729	2.042 bp in ATC210 corresponding to VP1575-VP1583, genes of prophage alpha	P	P	P	P	P	P	P	-
2,214,140–2,214,156	Not annotated in RIMD 2210633.	P	P	P	-	P	P	P	P
2,301,625–2,301,708	27 bp in VP2191	P	P	P	-	P	P	P	P
2,697,003–2,698,067	1064 bp in VP2552, DNA mismatch repair protein MutS	P	P	P	P	P	P	-	P
Chromosome 2*									
991,612–991,642	8-31 bp VPA0953 biofilm-associated surface protein	P	P	P	-	P	P	P	-

*Positions indicated in column Segment correspond to the larger uncovered segment.

P: Presence of the segment.

Presence and properties of regions not present in every Chilean isolate’s genome.

The bulk of the gained DNA, however, is plasmid located. Two isolates contain the previously described linear plasmid prophage VP58.5 [15], with no detectable high quality SNVs between them. One of these isolates (PMA109.5) contains besides VP58.5 two other large plasmids previously observed by gel electrophoresis of a plasmid extract (not shown). One of these plasmids (~82.0 Kb) is very similar to plasmid p0908, which was found in a *Vibrio* spp. and is similar to enterobacteria phage P1 [24]. The other plasmid (85.8 Kb) is highly similar to a plasmid (pVPUCMV) recently described in the environmental strain of *V. parahaemolyticus*, UCM-V493. Finally, there is a 361 bp contig containing a segment highly similar to *PvdJ*, a gene involved in pyoverdin synthesis in *Pseudomonas aeruginosa* PA381812 isolated from a patient. Pyoverdin competes directly with transferrin for iron and it is an essential element for *in vivo* iron gathering and virulence expression in *P. Aeruginosa*. This contig also contains segments identical to those in the RIMD 2210633 chromosome but it was not possible to identify it as an insert.

### Characteristics of RIMD 2210633 regions not covered by reads from at least one isolate

The larger of the regions (8,644 bp) absent in isolate PMC48 corresponds to the whole prophage f237 [23]. ATC210 has a deletion covering the region containing the capsid proteins of prophage alpha [25]. Reads from ATC210, PMC37.5, and PMC58.7 isolates did not cover a region of ~ 27 bp corresponding to VP2552 and part of VP2553, which comprise a hypothetical protein similar to the DNA mismatch repair protein MutS, and a DNA polymerase sigma factor, respectively.

## Discussion

Our results show that the *V. parahaemolyticus* pandemic strain population, usually called a clonal strain, constitutes an appropriate model to study the types and mechanisms of genome differentiation, a process responsible of the emergence of new pathogens. When comparing the genomes of these so-called genetically monomorphic organisms, we could distinguish differentiation by mutation producing SNVs and differentiation by genome content. The latter differences may be caused by DNA loss or gain. DNA gain occurs by HGT, a process well-established as a major driver of genetic innovation [26,27]. That HGT seems to be the major force shaping the virulence of *Vibrio* spp. was concluded by Chen et al. [11] after comparing the genomes of different *Vibrio* spp*.,* including three pandemic strains. By detailing here the number of SNVs among common regions of the genome and the amount of DNA gained or lost in the different isolates we found that both can largely differ between isolates (Table 2). We show that while SNVs among the Chilean isolates varies from 13 to 164, gained or lost DNA ranges from 1,366 to 217,729 bp. These results show that in pairwise comparisons (see by example PMA109.5 vs. PMA37.5) the changes in number of bases by HGT (gained or lost DNA, Table 2, under the diagonal) may be as high as about 5,400 times the number of SNVs (217,729/40) (Table 2, above the diagonal). A study on the evolutionary dynamics of *Vibrio cholerae* O1 following its introduction to Haiti [18], showed that intrinsic mutational processes accounted for virtually all the observed genetic polymorphism in the 23 genomes that were analyzed, with no demonstrable contribution from HGT. This situation is very similar to that observed with isolate ATC220, which did not contain DNA that was not present in any of the other Chilean isolates. Although the timespan of *V. cholerae* isolates is much shorter (less than one year), the apparent larger contribution of HGT to the genome differentiation of the Chilean *V. parahaemolyticus* isolates can be simply explained by the different ecological scenarios in which these different bacteria lived during the evolutionary time period that was studied. In the Haitian epidemics, propagation occurred mainly via contamination by fecal material containing *V. cholerae*, which reproduced in the human intestinal tract with little opportunity to meet other bacteria sufficiently related to effectively act as DNA donors, as would have occurred if significant growth had taken place in seawater environment. *V. parahaemolyticus* is not transmitted by fecal contamination and the isolates compared in this study reproduced in the littoral zone instead. Some, like PMA109.5 and PMC58.5, might have reproduced in a habitat experiencing close contact with a large and diverse population of other *V. parahaemolyticus* subgroups and *Vibrio* spp., while others, like ATC220, might have reproduced in a habitat free of *Vibrio* spp. sufficiently related to effectively act as DNA donors. When the pandemic strain grows in shellfish it shares this habitat with more than 60 different *V. parahaemolyticus* genotypes [7]. However, in contrast to what was observed in the Haitian epidemics, it is known that when *V. cholera* reproduces in open aquatic habitats HGT plays a large role in genome diversification [28]. Our results suggest that the predominant mechanism of genome diversification depends on the ecological scenario in which an isolate lived during the evolutionary period studied.

Nasser et al. [29] delineated the nature and timing of molecular events that contributed to other global human epidemic caused by an epidemic group with low synonymous sequence diversity, like pandemic *V parahaemolyticus*. They compared 3,615 genome sequences of group A *Streptococcus* differing strain-to-strain on average by only 48 SNPs, excluding the horizontally acquired polymorphisms in a 36-Kb region. Some of the difficulties to expect a similar successfully detailed description of the evolutionary pathway of pandemic *V. parahaemolyticus* to increased virulence by analysis of a larger number of isolates is that, differently to group A *Streptococcus V. parahaemolyticus* contains several natural hosts beside humans that there are not detailed analysis of their phenotypic properties related to pathogenicity.

The extraordinary number of SNVs in ATC210 might be the result of inherent properties of this strain, however, the possibility that the high number of SNVs in this isolate was simply due to a larger number of divisions because it benefited of a nutrient rich scenario is a simpler explanation. When dealing with short evolutionary times as in this case, the assumption accepted for long evolutionary periods that the replication rate between individuals is the same, is less likely and hence differences in the number of SNVs may correspond to differences in growth rate. If HGT depends on the number of divisions undergone by a bacterium, then a certain level of constancy might be expected between the amount of exclusive DNA (i.e., DNA absent from other isolates) and the number of SNVs present; however, to the contrary, this ratio was highly variable and fluctuated from 29 (ATC210/PMC58.7) to 5,443 (PMA109.5/PMA37.5).

The length of the RIMD 2210633 genome covered by the reads of all the isolates would correspond to the core genome of the group. This was calculated as 5,153,806 by subtracting those regions not covered by read from at least one isolate (Table 4) to the size of RIMD 2210633 genome. The sum of the length of the different DNA sequences not shared by all the isolates included in this study plus that of the core genome would correspond to the length of the pangenome of the group. This was estimated as 5,389,229 by adding to core genome those regions present in only some isolates (Table 4). Extra DNA was found on plasmids, temperate phages or inserted into the bacterial chromosomes. The 5,665 bp insertion found in every isolate examined except one has been described by Chen et al. [11] in the three pandemic strains they sequenced. Because no differences were found in the sequence and insertion site of the 5,665 bp IS among the pandemic isolates, its presence seems to be the result of a single event occurring early on the evolution of the pandemic strain. Presence of the 160/384 bp inserts in several isolates is a finding that is difficult to interpret. These inserts are similar to a segment described as being next to a transposase in plasmid p87F-2 from a new β-lactamase OXA-48 carbapenem resistance strain of *Enterobacter hormaechei* isolated in Brazil [30]. Here, the large DNA gain resulted from the presence of plasmid and temperate prophages. Their stability and effect on the recipient bacterium, especially its pathogenic character, is now a matter of speculation and further research. The extent of DNA loss was small compared with DNA gain and was mainly caused by complete or partial excision of the prophages. Excision of f237 has been described previously [31]. The f237 region is important because it contains ORF8, a region used extensively for differentiating pathogenic pandemic strains [32]. Consistent with recent concepts in bacterial genomics, the difference in genome content described above is an example of how rapidly pan-genomes can be created thereby diversifying an original clonal population in a short period of years.

## Conclusions

Populations of *V. parahaemolyticus* comprising pandemic strains are an appropriate model for studying the various types of genome differentiation and the mechanisms causing it despite the short evolutionary time frames involved. Our results show that while SNVs among the Chilean isolates varies from 13 to 164, gained or lost DNA varies from 1,366 to 217,729. Pairwise the number of bases changed by HGT (gained or lost DNA) may be from 30 to 5.400 higher than the number of SNVs, approximately. Since these isolates are genetically very similar, the different extent of SNVs and DNA gained or lost between isolates can be best explained by the life circumstances of each isolate.

## Methods

### Bacterial isolates and DNA extraction

The isolates used in this study have been described previously (Table 1). The RIMD 2210633 genome sequence [13] was used as a reference. VpKX corresponds to the RIMD 2210633 isolate from the Research Institute of Medical Disease in Japan (2002), thereafter maintained in our laboratory. Prior to sequencing, the bacteria were grown overnight in Luria Broth with vigorous agitation. DNA was extracted with a Wizard® Genomic DNA Purification Kit (Promega, Madison, WI).

### Genome data acquisition and initial data processing

DNA sequencing was performed in Ion Torrent for single-end using 100 bp chemistry libraries (Life Technologies, Carlsbad, CA) and in 454 FLX+ for mate paired-end libraries with a 3,000 bp span (Roche), for every isolate except PMA37.5. This last isolate was sequenced exclusively in Ion Torrent using library for single-end and 400 bp chemistry. Libraries preparation and sequencing were performed following the respective manufacturers instructions. For analysis of the sequences, the single-end raw reads from the Ion Torrent were converted into FASTQ format using sff2FASTQ software (http://github.com/indraniel/sff2FASTQ). Mate paired-end raw reads from FLX+ were converted into FASTQ format using sff_extract (http://bioinf.comav.upv.es/sff_extract/) and the following three files were generated: first mate paired-reads, second mate paired-reads and mate paired-end single reads without their pairs. The first and second mate paired-reads were trimmed with Trimmomatic v0.30 [33] using a Phred score of 15 (Q15), a 10 bp sliding window and a minimal read length of 35 bp. This process resulted in four files containing first-mate paired and second-mate paired reads after trimming, along with the single left and right reads generated by the loss of one of the pair after trimming. Files containing single-end reads (single left and right reads generated by loss of one of the pair after trimming, single-end raw reads, and mate paired-end single reads without a pair) were merged and trimmed by Trimmomatic with Q15, a 10 bp sliding window, and minimal read length of 70, thus generating one trimmed single-end file. This file constitutes the single-end reads used throughout this work.

### Variant calling

For variant calling, the raw sequence reads were imported into CLC Bio Genomics Workbench v6.0.4 (CLC Inc., Aarhus, Denmark) and mapped against the RIMD 2210633 reference sequence with 80% identity and a length fraction setting of 0.5. Variant calling was performed using the Probabilistic Variant Detection tool and adjusting the options specified in CLC to the parameters used for calling high quality variants of *V. cholerae* [18]. The parameters comprised SNV coverage of >10, variant probability of 75%, and ploidy set at 1. SNVs were subsequently subjected to a final manual review of the alignment graphics versus the variant calls for the nine genomes. SNVs between isolates were determined by aligning the sequence containing every position where a polymorphism was observed among the isolates. The cladogram was obtained using neighbor joining algorithm with Jukes-Cantor distance measure using 1,000 bootstrap replicates.

### Genome size estimation

Trimmed single-end and mate paired-end reads were aligned against the reference genome using Tmap (http://github.com/iontorrent/TMAP) with Map4 algorithm without soft-clipping. This allowed the length of the reference genome covered by the reads to be calculated. The size of the genomic DNA in an isolate other than the reference genome was estimated from the unmapped reads stored in a single BAM (http://samtools.sourceforge.net/SAM1.pdf) file for each library; these were extracted using SAMtools (http://samtools.sourceforge.net/), which identifies unmapped reads with the 0x4 flag. These reads were then processed by Perl regex ~ s/(\/1|\/2)//g to identify and delete from the end of the read identifiers (ids) containing the characters ‘/1′ or ′/2′, the Unix Sort command to alphabetically sort the read ids, the Unix Uniq command to select only one read id per pair, and a Python script called get_reads_by_id.py to obtain the initial reads from the trimmed FASTQ files. Reads treated in this manner were processed using FASTX Toolkit v0.0.13.1 (http://hannonlab.cshl.edu/fastx_toolkit/) to convert the first read pair from backwards to forwards to get the innie (forward-backward) orientation. Selected reads were finally assembled using the CELERA assembler (wgs assembler v7.0 [34]) and an insert size of 2,500 bp with a 500 bp standard deviation for mate paired-end reads, which generates scaffolds and degenerate contigs. Degenerate contigs were subsequently extended using CAP3 [35]. GC% was calculated using INFOSEQ (http://emboss.bioinformatics.nl/cgi-bin/emboss/infoseq). Assembled unaligned reads were uploaded with the following names: a) < isolate name > _Unaligned_Scfxxxx where the four “x” corresponded to the last four numbers assigned by the assembler, b) < isolate name > _Unaligned_Deg_Extxxxx, and c) < isolate name > _Unaligned_Degxxxx. The uploaded assembled reads were generated according to CELERA definitions (http://wgs-assembler.sourceforge.net/wiki/index.php/Celera_Assembler_Terminology). Coverage depth for assembled contigs was obtained using the fragmentDepth (−scaffold option) utility from the CELERA package. Degenerate contigs originating from the vectors used for library preparation and sequencing were removed using Blast VecScreen (http://blast.ncbi.nlm.nih.gov, using the UNIVEC database), after confirmation of the presence of the vectors’ multiple restriction site segments (polylinkers) from “Dynamic restriction site” in CLC. Degenerate contigs smaller than 316 bp, or with coverage depth lower than 11, were also filtered out of the analysis using a Perl command line. The size of a particular genome was estimated as the sum of the reference genome covered by the reads, plus the length of the scaffolds, contigs, or degenerate contig obtained for each isolate. Identification of regions in the reference genome not covered by reads was obtained by aligning the reads against the reference genome using the following two procedures: a) Tmap (as described above), SAMtools, and a custom script to identify regions with zero coverage and, b) CLC workbench (with 80% identity and 50% length fraction). Segments with zero coverage after conducting both procedures were identified as putative deletions. Inserts were identified by the presence of short sequences in their ends aligning with the reference genome. Additionally, their position was confirmed by identification and alignment of reads containing both the sequence of the reference genome and those in the contig that did not align with the reference. The following nucleotide sequences have been submitted to the NCBI BioProject PRJNA233509: Biosamples SAMN02781333, Pandemic *V. parahaemolyticus* ATC210; SAMN02781334, Pandemic *V. parahaemolyticus* ATC220; SAMN02781335, Pandemic *V. parahaemolyticus* PMA109.5; SAMN02781336, Pandemic *V. parahaemolyticus* PMC14.7; SAMN02781337, Pandemic *V. parahaemolyticus* PMC48; SAMN02781338, Pandemic *V. parahaemolyticus* PMC58.5; SAMN02781339, Pandemic *V. parahaemolyticus* PMC58.7; SAMN02781340, Pandemic *V. parahaemolyticus* VpKX; SAMN02781341, Pandemic *V. parahaemolyticus* PMA37.5.

## Additional file

Additional file 1: Table S1. Coverage observed after aligning of trimmed reads against reference genome.
