# Supplementary material for: Genome diversification within a clonal population of pandemic Vibrio parahaemolyticus seems to depend on the life circumstances of each individual bacteria
Source: BMC Genomics. 2015 Mar 13;16(1):176. doi: 10.1186/s12864-015-1385-8 (PMC4359782; doi:10.1186/s12864-015-1385-8)
Supplement: Additional file 1: Table S1. — Coverage observed after aligning of trimmed reads against reference genome. [file 12864_2015_1385_MOESM1_ESM.docx]

**Additional file 1: Table S1**

**Table S1.** Coverage observed after aligning of trimmed reads against reference genome.

| **Strain** | **Average coverage depth single ends** | **Average coverage depth mate paired-ends** | **Length coverage**  **(% ref genome)** |
| --- | --- | --- | --- |
| ATC210 | 25,3 | 3,1 | 5.163.347 |
| ATC220 | 43,9 | 3,6 | 5.165.607 |
| PMA109.5 | 49,3 | 2,7 | 5.165.581 |
| PMA37.5 | 42,0 | 0,0 | 5.164.877 |
| PMC14.7 | 46,4 | 1,5 | 5.165.617 |
| PMC48 | 29,0 | 5,1 | 5.158.212 |
| PMC58.5 | 47,7 | 2,9 | 5.165.521 |
| PMC58.7 | 36,2 | 1,6 | 5.164.548 |
| VpKX | 41,7 | 4 | 5.165.637 |
